# Experimental Researches into a New Excretory Function of the Liver; Consisting in the Removal of Cholesterine from the Blood, and Its Discharge from the Body in the Form of Stercorine

**Published:** 1862-10

**Authors:** Austin Flint

**Affiliations:** Professor of Physiology and Microscopy in the Bellevue Medical College, New York; Microscopist to Bellvue Hospital


### EXPERIMENTAL RESEARCHES INTO A NEW EXCRETORY FUNCTION OF THE LIVER; CONSISTING IN THE REMOVAL OF CHOLESTERINE FROM THE BLOOD, AND ITS DISCHARGE FROM THE BODY IN THE FORM OF STERCORINE. (*The Seroline of Boudet.*)

BY AUSTIN FLINT, JR., M.D., Professor of Physiology and Microscopy in the Bellvue Medical College, New York; Microscopist to Bellvue Hospital.

"*La Cholesterine du sang est elle un de ces produits destines a etre expulses de l'economie, et, par consequent, depourvus d'action immediate sur l'economie elle meme? Sa destination est tout a fait inconnue.*" Traite de Physiologie, par F. A. Longet. Paris, 1861. Tome i. p. 488.

This sentence, which was taken from the most elaborate treatise on physiology in any language, published in the centre of physiological science, in 1861, expresses the state of our knowledge with regard to the function of cholesterine. Cholesterine was discovered in 1782, by Poulletier de la Salle, in biliary calculi, and was detected upwards of thirty years ago, in the blood by Denis; but since then, with the exception of researches of a purely chemical nature into its properties, our knowledge with regard to it has not advanced. Its chemical history even, is far from perfect; while its physiological history is unknown. In 1833, Boudet discovered a substance in the blood which he called Seroline; a principle having many characters in common with cholesterine, but heretofore interesting merely as a curious proximate principle, found in excessively minute quantities in the serum of the blood only (whence its name); too minute, indeed, for ultimate analysis. Its function was as obscure as that of cholesterine. In examtning the literature of these two substances, we find that cholesterine is frequently not treated of in systematic works on physiology. Seroline is seldom even mentioned. Their function has been so obscure and apparently so unimportant, that theories with regard to it have not been advanced, and the highest chemical authorities, in speaking of their office in the economy, simply say of one, as of the other, that it is unknown. In the *Chimie Anatomique*, by Robin and Verdeil, we find cholesterine summed up in these words:—

"*Le role physiologique qu'elle remplit dans l'economie est egalement inconnu.*"

Of Seroline, the same authors say:—

"*On ne sait pas comment se forme la seroline, ni quel est son role physiologique.*"

Though the physiology of these substances is thus obscure, though chemistry has thus far done little for their history, and physiology nothing, certain facts with relation to them would seem to indicate that they are not unimportant in the economy. Cholesterine is found in the blood, bile, liver, nervous matter, crystalline lens, neconium (not in the feces, as incorrectly stated by authors), besides in a number of morbid products. It is found in these situations *constantly;* it appears in the bloood as soon as that liquid is found, and continues till the end of life. Its quantity in the blood is increased in certain diseased conditions, and diminished in others. Seroline has been said to exist constantly in the blood, though, till now, it has never been discovered in any other situation. It, like cholesterine, is a constant principle, and having many chemical characters in common. Their function is definite; it is important; and, if the writer do not exaggerate this importance in the enthusiasm of exploring a hitherto absolutely uncultivated field, a knowledge of the functions of these substances will be of incalculable value to the practical physician; and the path thus opened by physiology will lead to a great field for pathological inquiry. *What the discovery of the function of urea has done for diseases which now come under the head of uremia, the discovery of the function of Cholesterine may do for the obscure diseases which may hereafter be classed under the head of Cholesteremia.*

It is not surprising that the function of substances—which have been isolated with great difficulty, *which have never been found in any of the excretions*, which exist in quantity so small, that their investigation seemed to belong especially to the chemist, physiologists having been discouraged, perhaps, from studying them—should be thus obscure. But it is surprising that that important fluid, the bile, the product of the largest gland in the economy, and one of the most constantly found in the animal scale, should be so little understood. This has been regarded by some as a simple excrement, and by others as not an excrementitious, but a digestive fluid, and so much labor has been expended by physiologists in endeavours to settle this point, that no one has pretended to give an account of its excrementitious function, if it have any, and researches into its digestive function have left us almost entirely in the dark. Blondlot reported an observation on a dog which lived for five years with a biliary fistula diverting, as it is stated, all the bile from the intestines and discharging it from the body. The animal presented no untoward symptoms, died a natural death, no bile found its way into the intestines, but it was all discharged. According to this observation, the bile would appear to be purely an excrement, Schwann, and Bidder and Schmidt, in a large number of experiments, never succeeded in keeping a dog operated on in this way for more than a few weeks; they all died with evidences of inanition. The bile, according to these observations, is concerned chiefly in nutrition; and as it is poured into the upper part of the digestive tube, it is important, probably, in digestion. But Bidder and Schmidt do not satisfy us what its digestive function is; nor does Blondlot say what principle is excreted by it, nor what would be the result of its suppression.

Aside from a few facts, interesting enough, but indicating nothing definite, this is all we know of the function of the bile. But what physiologist does not feel this hiatus in his science; or what practical physician does not feel and know the importance of the function of the bile! It needs no inquiry into natural history, showing the universality, almost, of the liver in the animal scale, to impress upon the physician at the bedside the importance of the bile. A patient is suffering under an obscure ailment, which he may call biliousness or derangement of the liver, and which, in some unexplained way, is relieved by a mercurial purge. The practitioner knows that the bile-secreting function of the liver is important, but does not learn it from the physiologist. Every practitioner must feel that the liver has a function, which must be explained him by the physiologist, before he can avoid treating a large class of diseases empirically.

The bile has an important excretory function, which is liable to many disorders; and this function the writer hopes to be able, in the present article, to describe.

It will be seen by the preceding remarks that the physiological history of the bile remains to be written. The subject is too interesting and important not to engage the mind of the experimental physiologist. It is difficult at first sight to harmonize statements, to which reference has just been made, of experimenters, equally entitled to consideration, which are diametrically opposed. But of course the philosophical method of studying the bile is first to settle whether it be excrementitious or recrementitious. If the former, what substance is excreted, and where is it formed? If the latter, what function does it perform in any of the processes of nutrition. With the view to harmonize, if possible, in my own mind, the opposite statements of Bidder and Schmidt, and Blondlot, I attempted some time ago to establish biliary fistulæ in dogs. The first experiments were made in New Orleans, in the winter of 1860–61; but were all of them unsuccessful, no animal surviving the operation more than three days. The experiments were discontinued at that time, but were renewed in the winter of 1861–62, at the Bellevue Hospital Medical College. After a number of trials, which were no more successful than those made the previous winter, I succeeded in performing the operation with considerable rapidity and with very little disturbance of the abdominal organs, and in one animal the success was complete.

*Exp.* 1. The operation was performed by making an incision into the abdomen in the median line just below the ensiform cartilage, about three inches in length. The edge of the liver was carefully raised, the bile duct isolated, and two ligatures applied, one next the duodenum and the other near the junction of the ductus choledochus with the cystic duct, the intermediate portion being excised. The fundus of the gall-bladder was then drawn to the upper part of the wound, an incision made in it of about an inch in length, the bile evacuated, and the edges attached to the skin by points of the interrupted suture. The wound was then carefully closed around the opening into the gall-bladder.

This is nearly the proceeding recommended by Blondlot, who prefers, however, to operate while the animal is fasting, as the gall-bladder is then distended and can be more easily found. I have preferred to operate after feeding, when the gall-bladder is comparatively empty, as there is no great difficulty in finding it, and in evacuating its contents less bile is apt to find its way into the peritoneal cavity, which is one of the causes of the intense peritonitis which follows this operation.

The animal ate well the day after the operation, the bile flowed freely from the fistula and was entirely cut off from the intestine, as shown by *post mortem* examination. No symptoms supervened except those produced by the diversion of the bile from its normal course. This operation was performed on the 15th of November, 1861, and the animal lived thirty-eight days.

In no observation that I have found recorded has the animal been so free from inflammation consequent upon so serious an operation; and this seemed a most favorable opportunity of determining whether an animal could live with the bile shut off from the intestinal tube and discharged by a fistula. In this case the animal gradually lost flesh and strength, his appetite becoming voracious, until finally he died of inanition; the observation agreeing in every important particular with the experiments of Schwann, and Bidder and Schmidt.

*Exp.* 2. This experiment was undertaken to ascertain, if possible, the entire quantity of bile secreted in the twenty-four hours. A fistula was made into the ductus communis choledochus, the duct being divided and a silver tube introduced. The experiment did not succeed in point of view in which it was undertaken, and about forty-eight hours after the operation, the tube dropped out. After the removal of the tube the bile ceased to flow externally, and the animal did not appear to suffer any bad effects from the experiment. Thirty days after the operation, the animal having entirely recovered, he was killed by section of the medulla oblongata, and the parts carefully examined. The *post mortem* examination I transcribe from my note-book.

"On *post mortem* examination the liver was found adherent to the diaphragm over the greater part of its convex surface. There were evidences of limited inflammation over the duodenum. The liver itself was normal. Upon opening the duodenum, the papilla which marks the opening of the ductus communis choledochus was normal in appearance. A small silver stilet was introduced into the duct. *For a long time it was impossible to find any communication between the upper part of the duct and the intestine; but at last, after patient searching* (*knowing that no bile was discharged from the body, and that it was absolutely certain that a communication existed with the duodenum*), *a communication was found.* In Blondlot's case there probably was a communication re-established which escaped his observation."

In the remarkable observation reported by Blondlot, in which the animal survived for so long a period, the success is attributed to the fact that the dog was prevented from licking the bile as it flowed from the fistula, Blondlot stating that as soon as the animal was prevented from licking the bile, nutrition began to improve. Anxious to carry out all the precautions which had been adopted, I so muzzled the animal in Exp. 1, covering the lower part of the muzzle with oiled silk, that it was impossible for him to swallow a drop of the bile. This muzzle was kept on till the death of the animal, but the proceeding had no effect on his nutrition. The bile flowed so freely from the fistula that all the lower part of the animal was covered with it. It was not, however, until I made the *post mortem* examination in the second experiment that I was able to see the difficulty which I had experienced in harmonizing the observations of the different experimenters I have quoted. In the lower animals—in dogs, at least—ducts have a remarkable tendency to re-establish themselves. Any one who has operated much upon the glands can hardly fail to have noticed this fact. The pancreatic duct, for example, after having been divided and a tube introduced, becomes invariably re-established after the simple removal or dropping out of the tube. It was so with Exp. 2, in which the tube dropped out of the bile duct. The duct undoubtedly became re-established, for no bile flowed externally for nearly a month, the animal enjoying perfect health, and the fluid necessarily being emptied into the intestine; yet it was with the greatest difficulty that the communication could be found with the probe, and it was only after a long searching, knowing that there must be communication, that it was discovered at all. Taking into consideration the great difficulty I had in finding the passage in this instance, and after having carefully examined the case reported by Blondlot, I have concluded that a communication existed in his experiment which escaped observation, but by means of which a large quantity of the bile found its way into the intestine.*

With regard to the digestive function of the bile, it is sufficient to state here that the experiments which I have made on this subject have led me to believe that this fluid has an important office in connection with the function of digestion—one, indeed, which is essential to life. The nature of its office, however, is not understood, and can only be settled by a long

* An account of this experiment is to be found in an article entitled "*Essai sur les Fonctions du Foi et de ses annexes par N. Blondlot.*" 1846. The *post mortem* examination of the animal, made more than five years after the establishment of the fistula, was published in a little memoir complementary to the preceding, entitled "*Inutilite de la Bile dans La Digestion.*" 1851. It was not contemplated to enter into a full discussion of the views of Blondlot and others on the uses of the bile in digestion. That subject will be taken up in another paper in which the digestive properties of the bile will be mainly considered. In this connection it is proposed to take up only the excrementitious function of the bile.

and carefully executed series of experimental researches which would probably involve the whole subject of digestion. This I hope to be able to present in another paper. There is, however, another function of the bile entirely distinct from the preceding. It is the separation from the blood of the cholesterine, an excrementitious substance, which is formed by the destructive assimilation of certain tissues of the body. Though not discharged from the body as cholesterine, it being first changed into another substance, it is separated in that form from the blood and poured into the intestine by the ductus communis choledochus. This new excretory function of the bile will form a great part of the subject of this paper; the recrementitious function, which is necessary to complete the physiological history of this fluid, being deferred.

We will find the cholesterine to be the most important excrement separated by the liver, as the urea is the most important one separated by the kidneys; and the study of this substance will necessarily involve the depurative function of the liver. I will therefore begin with the cholesterine, and endeavor to show where it is formed in the economy, by following the blood in its passage through various organs. This will necessarily involve a description of the chemical processes which have been employed in its extraction. I will then endeavor to show where the cholesterine is removed from the blood, by the same method of investigation. The next step will be to follow it out of the body, and study the change which it undergoes in its passage through the alimentary canal. Having described the process of formation in the tissues, separation from the blood by the liver, and final discharge from the body, I will endeavor to show, finally, the effects of interruption of this function of the liver upon the economy. This will lead us into pathology, and a host of diseases will arise which may be dependent on a disturbance of the excretory function of the liver. We will be enabled to draw the line more closely between conditions in which there is resorption simply of the innocuous coloring matter of the bile, and those diseases in which there is a failure to separate the excrements from the blood. These conditions, it is well known, are widely different as to gravity, and the distinction between them is of great importance. The latter condition, characterized by the retention of cholesterine in the blood, will be treated of under the name of *Cholesteremia.*

CHOLESTERINE.

*Chemical characters.*—Cholesterine is a non-nitrogenized substance, having all the properties of the fats, excepting that of saponification with the alkalies. Its chemical formula is usually given as $C^{25}H^{22}O$. It belongs to a class of fatty substances which are non-saponifiable, which have been grouped by Lehmann under the name of lipoids. This class is composed of cholesterine and seroline, which are animal substances; castorine, from the *castoreum*, and amberin, from amber. To this he adds a substance discovered in a uterine tumor by Busch, called inosterine. Cholesterine is neutral, inodorous, crystallizable, insoluable in water, soluable in ether, very soluable in hot alcohol, though sparingly soluable in cold. It burns with a bright flame, but is not attacked by the alkalies, even after prolonged boiling. When treated with strong sulphuric acid, it strikes a peculiar red color, which is mentioned by some as characteristic of cholesterine. I have found that it possesses this character in common with seroline.*

*Forms of its crystals.*—Cholesterine may easily and certainly be recognized by the form of its crystals, the characters of which can be made out by means of the microscope. They are rectangular or rhomboidal, exceedingly thin and transparent, of variable size, with distinct and generally regular borders, and frequently arranged in layers with the borders of the lower ones showing through those which are superimposed. This arrangement of the crystals takes place when the cholesterine is present in considerable quantity. In pathological specimens they generally are few in number, and isolated. The plates of cholesterine are frequently marked by a cleavage at one corner, the lines running parallel to the borders; frequently they are broken, and the line of fracture is generally undulating. Lehmann attaches a great deal of importance to measurements of the angles of the rhomboid; according to this author, the obtuse angles are 100° 30′, and the acute 79° 30′. I have lately examined a great number of specimens of cholesterine, extracted from the blood, bile, brain, liver, and occurring in tumors, and am confident that the crystals have no definite angle. Frequently the plates are rectangular, and sometimes almost lozenge-shaped. It is by the transparency of the plates, the parallelism of their borders, and their tendency to break in parallel lines, that we recognize them as formed of cholesterine.

* This reaction of the seroline is mentioned by Berard, in the "*Cours de Physiologie*," tomme iii. p. 117.

Lehmann seems to consider the tablets of this substance as regular crystals, having invariable angles. From examination during crystallization, I am disposed to think that they are not crystals, but fragments of micaceous sheets, which, from their extreme tenuity, are easily broken. In examining a specimen from the meconium, which I extracted with hot alcohol, I was able to see a transparent film forming on the surface of the alcohol soon after it cooled; this, on microscopic examination, *in situ*, disturbing the fluid as little as possible, was found to be marked by long parallel lines. When the fluid had partially evaporated, it became broken and took the form of the ordinary crystals of cholesterine, but they were larger and more regular. The beauty of the tablets at this stage could not be adequately represented. They were exceedingly thin, and regularly divided into delicate plates, with the characteristic corner cleavages of the cholesterine; and, as the focus of the instrument was changed, new layers, with different arrangement, were brought into view. I have attempted to give an idea of the form of these tablets in Fig. 1; but it is, of course, impossible to represent their pale, but beautifully distinct borders. As has been remarked by Robin, the borders of these crystals can but be imperfectly imitated by a line; there is no line in the object itself, but the edge shows where the tablet ceases. (See Fig. 1.)

The crystals are generally colorless, but when present in colored fluids, may take a yellowish tint, or even become very dark. They may still be recognized, however, by the characters of form just described.

Crystals of cholesterine melt at 293° Fahr., but are formed again when the temperature falls below that point. According to Lehmann, they may be distilled *in vacuo* at 680° without decomposition. The determination of the fusing point is one of the means of distinguishing it from seroline, which fuses at 90° 8′.

*Situation of the Cholesterine.*—Most authors state that the cholesterine is found in the bile, blood, liver, brain, and nerves, crystalline lens, meconium, and the fecal matter. I have found the cholesterine in all these situations invariably, excepting the feces, where it was seen but once after a number of examinations; and in studying the works of those who have investigated this substance, I can find no one who has found it in the normal feces. It is found in large quantities in the meconium, from which, perhaps, it is most easily extracted in a state of purity, and has been extracted from the feces of animals in a state of hibernation; but though it may occasionally be found in the feces in disease, and in animals after long fasting, I am confident that it never occurs in the ordinary conditions. The analysis of the fecal matter is so unattractive, that it has been very much neglected by chemists; and until a few years ago, when an elaborate analysis was made by Marcet, to which reference will hereafter be made, the analysis of Berzelius formed nearly all our data on this subject. Cholesterine forms the greater part of biliary calculi, which indeed consists generally of nothing but cholesterine, coloring matter, and mucus. It is found in a large number of morbid deposits. Few cases of cancer are examined without discovering tablets of cholesterine. It is very abundant in encysted tumors. According to Robin, atheromatous deposits, which are found in the middle coats of the arteries, are often composed of cholesterine. It sometimes forms distinct tumors or deposits in the substance of the brain. I lately had an opportunity of examining a tumor from the brain, at the Bellevue Hospital, which consisted of nearly pure cholesterine. It has often been found in a fluid of hydrocele, in the fluid of ovarian cysts, in crude tubercle, in epithelial tumors, and in pus. The proportion in which it exists in the fluids of the body is very small. I have made a number of quantitative analysis of the blood, the results of which I give in the following table, with some of the analysis which have been made for this substance. I also give the quantity which I have found in the other situations in which it is found. The variations in different parts of the circulation and in diseased conditions will be given in another table. The quantity in the brain and crystalline lens has, I believe, never before been estimated:—

*Table of Quantity of Cholesterine in various Situations.*

| Situation. | Observer. | Quantity examined. | Cholesterine per 1,000 pts. |
|---|---|---|---|
| Venous blood (male) | Becquerel and Rodier. | | 0.090 |
| Do. (female) | Becquerel and Rodier. | *grains.* | 0.090 |
| Do. (male æt. 35.) | A. Flint, Jr. | 312.083 | 0.445 |
| Do. (male æt. 22.) | A. Flint, Jr. | 187.843 | 0.658 |
| Do. (male æt. 24.) | A. Flint, Jr. | 102.680 | 0.751 |
| Bile (human) | Frerichs. | | 1.600 |
| Do. (normal of ox) | Berzelius. | | 1.000 |
| Do. (human) | A. Flint, Jr. | 224.588 | 0.618 |
| Meconium. | Simon. | | 160.000 |
| Do. | A. Flint, Jr. | 170.541 | 6.245 |
| Brain (human) | A. Flint, Jr. | 159.753 | 7.729 |
| Do. do. | A. Flint, Jr. | 150.881 | 11.456 |
| Crystalline lens (ox)* | A. Flint, Jr. | 135.020 | 0.907 |

* In this examination four fresh crystalline lenses of the ox were used.

*Form under which the cholesterine exists in the organism.*—In the fluids of the body cholesterine exists in a state of solution, but by virtue of what constituents it is held in solution, is not entirely settled. It is stated that the biliary salts have the power of holding it in solution in the bile, and that the small amount of fatty acids which are contained in the blood hold it in solution in that fluid, but direct experiments on this point are wanting. In the nervous substance and in the crystalline lens it is united "*molecule a molecule*" to the other elements which go to make up these tissues. After it is discharged into the intestinal canal, when it is not changed into stercorine, it is to be found in a crystalline form, as in the meconium, and in the feces of animals in a state of hibernation. In pathological fluids and in tumors, it is found in a crystalline form, and may be detected by microscopic examination.

*Process for the extraction of cholesterine.*—Without describing the processes which have been employed by other observers for the extraction of cholesterine from the blood, bile, and various tissues of the body, I will confine myself to a description of the process which I have found most convenient to employ in the analysis I have made for this substance. In analysis of gall-stones the process is very simple; all that is necessary being to pulverize the mass, and extract it with boiling alcohol; filter the solution while hot, the cholesterine being deposited on cooling. If the crystals be colored, they must be redissolved, and filtered through animal charcoal. This is the proceeding employed by Poulletier de la Salle, Fourcroy, and Chevreul. It is only when this substance is mixed with fatty matters, that its isolation is a matter of any difficulty. In extracting cholesterine from the blood, I have operated on both the serum and clot, and in this way have been able to demonstrate it in greater quantities in this fluid than has been observed by others, who employed only the serum. The following is the process for quantitative analysis, which I determined upon after a number of experiments.

The blood, bile, or brain, as the case may be, is first carefully weighed, then evaporated to dryness over a water bath, and carefully pulverized in an agate mortar, so as to collect every particle. The powder is then treated with ether, in the proportion of about a fluidounce for every hundred grains of the original weight, for from twelve to twenty-four hours, agitating the mixture occasionally. The ether is then separated by filtration, throwing a little fresh ether on the filter so as to wash through every trace of the fat, and the solution set aside to evaporate.* If the fluid, especially the blood, have been carefully dried and pulverized, when the ether is added it divides into a very fine powder, and penetrates every part. After the ether has evaporated, the residue is extracted with boiling alcohol, in the proportion of about a fluidrachm for every hundred grains of the original weight of the specimen, filtered, while hot, into a watch-glass, and allowed to evaporate spontaneously. To keep the fluid hot while filtering, the whole apparatus may be placed in the chamber of a large water-bath, or, as the filteration is generally rapid, the funnel may be warmed by plunging it into hot water, or steaming it, taking care that it be carefully wiped. We now have the cholesterine mixed with a certain kind of saponifiable fat. After the fluid has evaporated, we can see the cholesterine crystallized in the watch-glass, mingled with masses of fat. This we remove by saponification with an alkali; and for this purpose, we add a moderately strong solution of caustic potash, which we allow to remain in contact with the residue for from one to two hours. If much fat be present, it is best to subject the mixture to a temperature a little below the boiling point; but in analysis of the blood, this is not necessary. The mixture is then to be largely diluted with distilled water, thrown upon a small filter, and thoroughly washed till the solution which passes through is neutral. We then dry the filter, and fill it up with ether, which, in passing through, dissolves out the cholesterine. The ether is then evaporated, the residue extracted with boiling alcohol, as before, the alcohol collected on a watch-glass, previously weighed, and allowed to evaporate, The residue consists of pure cholesterine, the quantity of which may be estimated by weight.

The accuracy of this process may be tested by means of the microscope. As the crystals have so distinctive a form under the microscope, it is easy to determine by examining the watch-glass, whether it has been obtained in a state of purity. In making this analysis quantitatively, it is necessary to be very careful in all the manipulations; and for determining the weight of such minute quantities, an accurate and delicate balance, one, at least, that will turn with the thousandth of a gramme, carefully adjusted, must be employed. With these precautions, the quantity of cholesterine in any fluid or solid may be determined with perfect accuracy. The quantity of cholesterine may be estimated in from fifteen to twenty grains of blood. In analyzing the brain and bile, I found it necessary to pass the first

* The ether may be preserved by distillation, instead of allowing it to evaporate, but the small quantity usually employed this is hardly worth while.

ethereal solution through animal charcoal, to get rid of the coloring matter. In doing this, the charcoal must be washed with fresh ether till the solution which passes through is brought up to the original quantity. The other manipulations are the same as in examinations of the blood. In examining the meconium, I found that the cholesterine which crystallized from the first alcoholic extract was so pure that it was not necessary to subject it to the action of an alkali.

I am aware that in describing the process for the extraction of cholesterine I have entered into details which would be superfluous for the practical chemist. But the extraction of this substance from the blood is so simple, and the results of the examination of the blood in different parts of the circulatory system have been so striking and important, that I cannot but indulge the hope that the observations which follow will be verified by those who may not be skilful practical chemists. Almost any one is competent to make a quantitative analysis of the blood for cholesterine. It simply requires six days for the process, and a number of analysis may be carried on at the same time. It requires one day, after the blood his been dried and pulverized, for the ether to act upon it; the next morning it is filtered and set aside; the next morning it will be dry, and may be extracted with alcohol, and set aside to evaporate; the next morning it may be treated with potash, filtered, and the filter washed with water; the following day it may be washed with ether, and set aside to evaporate; the following day it will have evaporated, and may be extracted with hot alcohol; and the following day the alcohol will have evaporated, and the specimen may be examined by the microscope and weighed. All that is required is a little care in the performance of these manipulations—which one with a slight acquaintance with operations in chemistry may perform at once, and one or two trials will enable a novice to execute—and accuracy in weighing, which is, indeed, the most delicate part of the process.

*History of cholesterine.*—A brief sketch of the history of this substance may not be uninteresting. It was first obtained by Poulletier de la Salle, in 1782, who extracted it from a biliary calculus. He communicated his observations to Fourcroy, who published them, after having verified his experiments, the death of the discoverer preventing him from making his observations public. Afterwards, in examining an old, hardened, liver, Fourcroy found a concrete, oily substance, analagous to that discovered by Poulletier. He imagined that the liver had become changed into a substance resembling spermaceti. The cholesterine was afterwards found in gall-stones, by Vicq d' Azyr, by Jaquin, Titus, and Kreysig. In 1791, Fourcroy described a substance which he called adipocire, found in bodies at the *cimetiere des Innocents*, which he likened to spermaceti and to cholesterine. He always, however, made a distinction between these substances; calling the cholesterine *crystallizable adipocire.* In 1814, Chevreul established the difference between the adipocire and the cholesterine, giving a full description of the cholesterine. He extracted it from the bile of the human subject, of the bear, and of the pig.

After that time a number of chemists found it in the gall-stones and intestinal concretions. Lassaigne found it in a cerebral tumor, Guerard in hydatid cysts of the liver, Morin in the liquid from an abdominal tumor, Caventou in the matter from an abscess under the malar bone, and a number of others in tumors in various situations. In 1830, it was discovered in the blood by Denis, and afterwards described by Boudet, who wrote an elaborate article on the composition of the serum of the blood in 1833, in which he describes the cholesterine and a new substance which he called *seroline.** It was also detected in normal blood by Lecanu and Marchand. Couerbe, who made elaborate researches into the chemical composition of the cerebral substance, pointed out the existence of cholesterine in the brain. Lebert found it in the substance of cancerous tumors, Curling found it in the fluid of hydrocele, Simon extracted it from the meconium, and Preuss discovered it in the substance of crude tubercle. Of late authors, Becquerel and Rodier have been most extended in their investigation of this principle.† They have made a number of careful quantitative analysis of the blood for this substance in health and disease. Their observations will be more particularly referred to further on.‡

[*Continued in the next Number.*]

* Boudet, Nouvelles Recherches sur la composition du serum du sang humain. Annales du Chimie et de Physique, tom. lii. p. 337.

† Traite de Chimie Pathologique appliquee a la medecin pratique, par M. Alf. Becquerel, professeur agrege, etc., et par M. A. Rodier, Docteur en Medecine, etc. Paris, 1854, and Recherches sur la composition du sang. Paris, 1844.

‡ The history of the cholesterine was mostly compiled from the excellent work of Robin and Verdeil, the Chimie Anatomique.

